# (*E*)-3-[(4-Butyl­phen­yl)imino­meth­yl]benzene-1,2-diol

**DOI:** 10.1107/S1600536809029316

**Published:** 2009-07-29

**Authors:** Zeynep Keleşoğlu, Orhan Büyükgüngör, Çiğdem Albayrak, Mustafa Odabaşoğlu

**Affiliations:** a Department of Physics, Ondokuz Mayıs University, TR-55139 Samsun, Turkey; b Sinop University, Sinop Faculty of Education, Sinop, Turkey; c Pamukkale University, Denizli Technical Vocational School, Denizli, Turkey

## Abstract

The title compound, C_17_H_19_NO_2_, exists as an enol–imine tautomer. The dihedral angle between the two benzene rings is 4.6 (2)°. The mol­ecular structure is stabilized by intramol­ecular O—H⋯O and O—H⋯N hydrogen bonds which generate *S*(5) and *S*(6) ring motifs, respectively. In the crystal, mol­ecules are linked into centrosymmetric dimers by pairs of O—H⋯O hydrogen bonds. In addition, C—H⋯π inter­actions involving both benzene rings are observed.

## Related literature

For general background to Schiff bases, see: Lozier *et al.* (1975 ▶); Calligaris *et al.* (1972 ▶); Maslen & Waters (1975 ▶); Steward & Lingafelter (1959 ▶). For the photochromic and thermochromic characteristics of Schiff base compounds, see: Hadjoudis *et al.* (1987 ▶); Moustakali-Mavridis *et al.* (1980 ▶). For graph-set motifs, see: Bernstein *et al.* (1995 ▶). For related structures, see: Temel *et al.* (2007 ▶); Koşar *et al.* (2005 ▶).
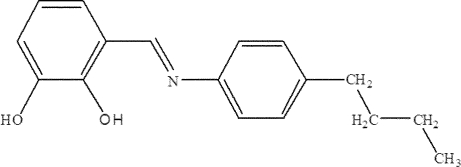


## Experimental

### 

#### Crystal data

C_17_H_19_NO_2_
*M* *_r_* = 269.33
Monoclinic, 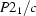

*a* = 16.2774 (13) Å
*b* = 6.0148 (6) Å
*c* = 17.6166 (14) Å
β = 121.476 (5)°
*V* = 1471.0 (2) Å^3^
*Z* = 4
Mo *K*α radiation
μ = 0.08 mm^−1^
*T* = 296 K
0.50 × 0.45 × 0.03 mm

#### Data collection

Stoe IPDSII diffractometer
Absorption correction: integration (*X-RED32*; Stoe & Cie, 2002 ▶) *T*_min_ = 0.954, *T*_max_ = 0.998
8711 measured reflections
3061 independent reflections
1643 reflections with *I* > 2σ(*I*)
*R*_int_ = 0.062

#### Refinement

*R*[*F* ^2^ > 2σ(*F* ^2^)] = 0.064
*wR*(*F* ^2^) = 0.163
*S* = 1.07
3061 reflections
189 parameters
2 restraints
H atoms treated by a mixture of independent and constrained refinement
Δρ_max_ = 0.15 e Å^−3^
Δρ_min_ = −0.15 e Å^−3^

### 

Data collection: *X-AREA* (Stoe & Cie, 2002 ▶); cell refinement: *X-AREA*; data reduction: *X-RED32* (Stoe & Cie, 2002 ▶); program(s) used to solve structure: *SHELXS97* (Sheldrick, 2008 ▶); program(s) used to refine structure: *SHELXL97* (Sheldrick, 2008 ▶); molecular graphics: *ORTEP-3 for Windows* (Farrugia, 1997 ▶); software used to prepare material for publication: *WinGX* (Farrugia, 1999 ▶).

## Supplementary Material

Crystal structure: contains datablocks I, global. DOI: 10.1107/S1600536809029316/ci2863sup1.cif

Structure factors: contains datablocks I. DOI: 10.1107/S1600536809029316/ci2863Isup2.hkl

Additional supplementary materials:  crystallographic information; 3D view; checkCIF report

## Figures and Tables

**Table 1:** Hydrogen-bond geometry (Å, °)

*D*—H⋯*A*	*D*—H	H⋯*A*	*D*⋯*A*	*D*—H⋯*A*
O2—H2⋯O1	0.86 (2)	2.21 (3)	2.728 (2)	118 (3)
O2—H2⋯O1^i^	0.86 (2)	2.08 (3)	2.802 (3)	141 (3)
O1—H1⋯N1	0.88 (2)	1.74 (2)	2.555 (2)	155 (3)
C6—H6⋯*Cg*2^ii^	0.93	2.85	3.645 (3)	144
C10—H10⋯*Cg*1^ii^	0.93	2.80	3.491 (3)	132

Symmetry codes: (i) 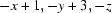; (ii) 
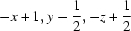
. *Cg*1 and *Cg*2 are the centroids of the C1–C6 and C8–C13 rings, respectively.

**Fig. 1.**
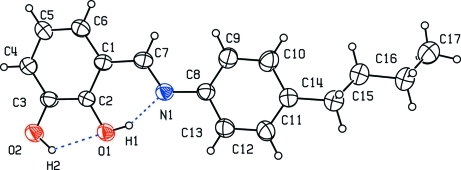
An ORTEP view of (I), with the atom-numbering scheme and 30% probability displacement ellipsoids. Dashed lines indicate H-bonds.

**Fig. 2.**
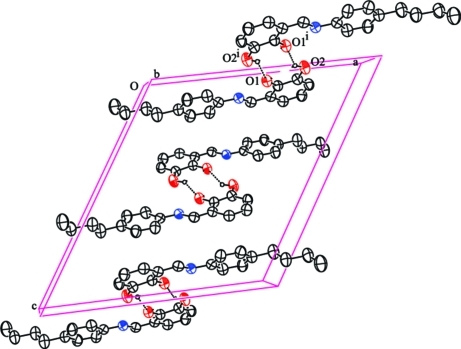
A packing diagram for (I), showing the formation of dimers through O—H···O hydrogen bonds. H atoms not involved in hydrogen bonding (dashed lines) have been omitted for clarity [symmetry code (i): 1-x, 3-y, -z].

## supplementary crystallographic information

### Comment

Schiff bases are widely used as ligands in the field of coordination chemistry and they play an important role in various field of chemistry due to their biological activities (Lozier *et al.*, 1975). *o*-Hydroxy Schiff bases derived from the reaction of *o*-hydroxy aldehydes with aniline have been examined extensively (Steward & Lingafelter, 1959; Calligaris *et al.*, 1972; Maslen & Waters, 1975). Some Schiff bases derived from salicylaldehyde have attracted the interest of chemists and physicists because they show thermochromism and photochromism in the solid state by H-atom transfer from the hydroxy O atom to the N atom (Hadjoudis, *et al.*, 1987). It has been proposed that molecules showing thermochromism are planar while those showing photochromism are non-planar (Moustakali-Mavridis *et al.*, 1980). There are two types of intramolecular hydrogen bonds in Schiff bases arising from the keto-amine (N—H···O) and enol-imine (N···H—O) tautomeric forms.

X-ray analysis shows that compound (I) prefers the enol-imine tautomeric form with a strong intramolecular O—H···N hydrogen bond. A H atom is located on atom O1, thus the enol-imine tautomer is favoured over the keto-amine form, as indicated by the C2—O1 [1.333 (2) Å], C7—N1 [1.297 (2) Å], C1—C7 [1.433 (2) Å] and C1—C2 [1.406 (2) Å] bond lengths (Fig. 1). The C2—O1 bond length of 1.333 (2) Å indicates a single-bond character, whereas the C7—N1 bond length of 1.297 (2) Å indicates a high degree of double-bond character. Similar results were observed for (*E*)-3-[(2-fluorophenylimino)methyl]benzene-1,2-diol [C—O = 1.354 (19) Å, C—N = 1.285 (2) Å; Temel *et al.*, 2007].

The molecule of (I) is nearly planar, with a dihedral angle between the benzene rings A(C1-C6) and B(C8-C13) of 4.6 (2) Å. Intramolecular O—H···O and O—H···N hydrogen bonds generate S(5) and S(6) ring motifs, respectively (Bernstein *et al.*, 1995) (Fig. 1). The nearly planar S(6) ring forms dihedral angles of 2.3 (4)° and 2.5 (5)° with the rings A and B, respectively.

In the crystal, molecules of (I) are linked by intermolecular O—H···O hydrogen bonds forming centrosymmetric dimers (Fig.2). In addition, C6—H6···*Cg*2 and C10—H10···*Cg*1 interactions (*Cg*1 and *Cg*2 are the centroids of the C1—C6 and C8—C13 rings, respectively) are observed (Table 1).

### Experimental

Compound (I) was prepared by refluxing a mixture of 2,3-dihydroxy benzaldehyde (0.5 g, 0.0036 mol) in ethanol (20 ml) and 4-butilanilyne (0.54 g 0.0036 mol) in ethanol (20 ml). The reaction mixture was stirred for 1 h under reflux. The crystals of (I) suitable for X-ray analysis were obtained from a methanol solution by slow evaporation (yield 85%; m.p. 363–364 K).

### Refinement

The hydroxyl H atoms were located in a difference Fourier map and were refined with a O-H distance restraint of 0.83 (2) Å. All other H-atoms were refined using a riding model with C-H = 0.93–0.96 Å (*U*_iso_ = 1.2*U*_eq_ of the parent atom) for aromatic and ethyl C atoms and C-H = 0.97 Å (*U*_iso_ = 1.5*U*_eq_ of the parent atom) for methyl C atoms.

### Crystal data

**Table:**

C_17_H_19_NO_2_	*F*(000) = 576
*M**_r_* = 269.33	*D*_x_ = 1.216 Mg m^−^^3^
Monoclinic, *P*2_1_/*c*	Mo *K*α radiation, λ = 0.71073 Å
Hall symbol: -P 2ybc	Cell parameters from 8711 reflections
*a* = 16.2774 (13) Å	θ = 1.4–27.4°
*b* = 6.0148 (6) Å	µ = 0.08 mm^−^^1^
*c* = 17.6166 (14) Å	*T* = 296 K
β = 121.476 (5)°	Thin plate, red
*V* = 1471.0 (2) Å^3^	0.50 × 0.45 × 0.03 mm
*Z* = 4	

### Data collection

**Table:**

Stoe IPDSII diffractometer	3061 independent reflections
Radiation source: fine-focus sealed tube	1643 reflections with *I* > 2σ(*I*)
graphite	*R*_int_ = 0.062
Detector resolution: 6.67 pixels mm^-1^	θ_max_ = 26.5°, θ_min_ = 1.5°
rotation method scans	*h* = −20→20
Absorption correction: integration (*X-RED32*; Stoe & Cie, 2002)	*k* = −7→7
*T*_min_ = 0.954, *T*_max_ = 0.998	*l* = −22→22
8711 measured reflections	

### Refinement

**Table:**

Refinement on *F*^2^	Secondary atom site location: difference Fourier map
Least-squares matrix: full	Hydrogen site location: inferred from neighbouring sites
*R*[*F*^2^ > 2σ(*F*^2^)] = 0.064	H atoms treated by a mixture of independent and constrained refinement
*wR*(*F*^2^) = 0.163	*w* = 1/[σ^2^(*F*_o_^2^) + (0.0648*P*)^2^ + 0.0507*P*] where *P* = (*F*_o_^2^ + 2*F*_c_^2^)/3
*S* = 1.07	(Δ/σ)_max_ = 0.044
3061 reflections	Δρ_max_ = 0.15 e Å^−^^3^
189 parameters	Δρ_min_ = −0.15 e Å^−^^3^
2 restraints	Extinction correction: *SHELXL97* (Sheldrick, 2008), Fc^*^=kFc[1+0.001xFc^2^λ^3^/sin(2θ)]^-1/4^
Primary atom site location: structure-invariant direct methods	Extinction coefficient: 0.0060 (18)

### Special details

**Table:**

Geometry. All e.s.d.'s (except the e.s.d. in the dihedral angle between two l.s. planes) are estimated using the full covariance matrix. The cell e.s.d.'s are taken into account individually in the estimation of e.s.d.'s in distances, angles and torsion angles; correlations between e.s.d.'s in cell parameters are only used when they are defined by crystal symmetry. An approximate (isotropic) treatment of cell e.s.d.'s is used for estimating e.s.d.'s involving l.s. planes.
Refinement. Refinement of *F*^2^ against ALL reflections. The weighted *R*-factor *wR* and goodness of fit *S* are based on *F*^2^, conventional *R*-factors *R* are based on *F*, with *F* set to zero for negative *F*^2^. The threshold expression of *F*^2^ > σ(*F*^2^) is used only for calculating *R*-factors(gt) *etc*. and is not relevant to the choice of reflections for refinement. *R*-factors based on *F*^2^ are statistically about twice as large as those based on *F*, and *R*- factors based on ALL data will be even larger.

### Fractional atomic coordinates and isotropic or equivalent isotropic displacement parameters (Å^2^)

**Table:**

	*x*	*y*	*z*	*U*_iso_*/*U*_eq_	
C1	0.57490 (18)	0.9538 (4)	0.13401 (17)	0.0578 (6)	
C2	0.57322 (18)	1.1547 (4)	0.09238 (17)	0.0572 (6)	
C3	0.64541 (18)	1.1943 (4)	0.07262 (18)	0.0613 (7)	
C4	0.71906 (19)	1.0448 (5)	0.0989 (2)	0.0690 (8)	
H4	0.7677	1.0749	0.0876	0.083*	
C5	0.7222 (2)	0.8493 (5)	0.1423 (2)	0.0733 (8)	
H5	0.7726	0.7496	0.1597	0.088*	
C6	0.65099 (19)	0.8032 (4)	0.15939 (18)	0.0666 (7)	
H6	0.6530	0.6715	0.1880	0.080*	
C7	0.49848 (19)	0.9019 (4)	0.14886 (18)	0.0621 (7)	
H7	0.4991	0.7663	0.1746	0.074*	
C8	0.34786 (18)	0.9936 (4)	0.13574 (17)	0.0586 (6)	
C9	0.3369 (2)	0.8029 (5)	0.1736 (2)	0.0760 (8)	
H9	0.3838	0.6926	0.1947	0.091*	
C10	0.2569 (2)	0.7761 (5)	0.1801 (2)	0.0787 (9)	
H10	0.2511	0.6477	0.2064	0.094*	
C11	0.1851 (2)	0.9339 (5)	0.1489 (2)	0.0697 (8)	
C12	0.1964 (2)	1.1213 (5)	0.1113 (2)	0.0789 (9)	
H12	0.1490	1.2305	0.0898	0.095*	
C13	0.2768 (2)	1.1527 (4)	0.1044 (2)	0.0732 (8)	
H13	0.2826	1.2819	0.0785	0.088*	
C14	0.0994 (2)	0.9044 (5)	0.1604 (3)	0.0939 (10)	
H14A	0.1223	0.9125	0.2234	0.113*	
H14B	0.0555	1.0278	0.1314	0.113*	
C15	0.0450 (2)	0.6941 (6)	0.1246 (2)	0.0910 (10)	
H15A	0.0880	0.5706	0.1557	0.109*	
H15B	0.0247	0.6822	0.0623	0.109*	
C16	−0.0432 (2)	0.6723 (6)	0.1326 (3)	0.0975 (11)	
H16A	−0.0230	0.6826	0.1949	0.117*	
H16B	−0.0862	0.7959	0.1018	0.117*	
C17	−0.0972 (3)	0.4600 (6)	0.0956 (3)	0.1137 (13)	
H17A	−0.1126	0.4420	0.0354	0.171*	
H17B	−0.1555	0.4640	0.0967	0.171*	
H17C	−0.0581	0.3375	0.1310	0.171*	
N1	0.42843 (15)	1.0402 (3)	0.12717 (14)	0.0599 (6)	
O1	0.50446 (13)	1.3071 (3)	0.06822 (13)	0.0666 (5)	
O2	0.64235 (14)	1.3817 (3)	0.02744 (15)	0.0767 (6)	
H1	0.466 (2)	1.247 (5)	0.083 (2)	0.115*	
H2	0.5901 (17)	1.449 (5)	0.016 (2)	0.115*	

### Atomic displacement parameters (Å^2^)

**Table:**

	*U*^11^	*U*^22^	*U*^33^	*U*^12^	*U*^13^	*U*^23^
C1	0.0654 (15)	0.0468 (13)	0.0619 (17)	−0.0011 (12)	0.0337 (14)	0.0022 (12)
C2	0.0608 (15)	0.0481 (13)	0.0663 (17)	−0.0007 (12)	0.0355 (14)	−0.0042 (12)
C3	0.0708 (16)	0.0471 (13)	0.0725 (19)	−0.0035 (12)	0.0421 (15)	−0.0018 (13)
C4	0.0678 (16)	0.0638 (16)	0.083 (2)	−0.0022 (14)	0.0445 (16)	−0.0101 (16)
C5	0.0736 (18)	0.0612 (17)	0.088 (2)	0.0099 (14)	0.0442 (17)	−0.0008 (16)
C6	0.0732 (17)	0.0527 (14)	0.0730 (19)	0.0058 (13)	0.0375 (15)	0.0042 (14)
C7	0.0723 (17)	0.0512 (14)	0.0642 (18)	−0.0018 (13)	0.0367 (14)	0.0041 (13)
C8	0.0630 (15)	0.0535 (14)	0.0608 (17)	−0.0019 (12)	0.0335 (13)	−0.0001 (13)
C9	0.0702 (17)	0.0635 (16)	0.097 (2)	0.0085 (14)	0.0454 (17)	0.0242 (17)
C10	0.0731 (17)	0.0723 (18)	0.096 (2)	0.0003 (15)	0.0476 (17)	0.0199 (17)
C11	0.0681 (17)	0.0650 (17)	0.081 (2)	0.0001 (14)	0.0426 (16)	−0.0011 (16)
C12	0.0754 (19)	0.0657 (17)	0.103 (2)	0.0118 (15)	0.0521 (19)	0.0094 (17)
C13	0.0798 (18)	0.0568 (15)	0.091 (2)	0.0072 (14)	0.0504 (17)	0.0138 (16)
C14	0.087 (2)	0.084 (2)	0.129 (3)	−0.0094 (18)	0.069 (2)	−0.016 (2)
C15	0.0775 (19)	0.091 (2)	0.117 (3)	−0.0056 (18)	0.059 (2)	−0.007 (2)
C16	0.084 (2)	0.107 (3)	0.121 (3)	−0.0090 (19)	0.066 (2)	−0.008 (2)
C17	0.106 (3)	0.095 (3)	0.161 (4)	−0.010 (2)	0.085 (3)	−0.002 (3)
N1	0.0647 (12)	0.0532 (12)	0.0649 (15)	−0.0010 (11)	0.0361 (11)	0.0026 (11)
O1	0.0737 (12)	0.0515 (10)	0.0877 (14)	0.0050 (9)	0.0512 (11)	0.0095 (10)
O2	0.0887 (14)	0.0557 (11)	0.1117 (17)	0.0022 (10)	0.0704 (14)	0.0086 (11)

### Geometric parameters (Å, °)

**Table:**

C1—C6	1.406 (3)	C10—H10	0.93
C1—C2	1.406 (3)	C11—C12	1.367 (4)
C1—C7	1.433 (3)	C11—C14	1.520 (4)
C2—O1	1.333 (3)	C12—C13	1.389 (4)
C2—C3	1.409 (3)	C12—H12	0.93
C3—O2	1.365 (3)	C13—H13	0.93
C3—C4	1.371 (4)	C14—C15	1.483 (4)
C4—C5	1.389 (4)	C14—H14A	0.97
C4—H4	0.93	C14—H14B	0.97
C5—C6	1.370 (4)	C15—C16	1.519 (4)
C5—H5	0.93	C15—H15A	0.97
C6—H6	0.93	C15—H15B	0.97
C7—N1	1.297 (3)	C16—C17	1.493 (5)
C7—H7	0.93	C16—H16A	0.97
C8—C13	1.375 (3)	C16—H16B	0.97
C8—C9	1.384 (3)	C17—H17A	0.96
C8—N1	1.424 (3)	C17—H17B	0.96
C9—C10	1.375 (4)	C17—H17C	0.96
C9—H9	0.93	O1—H1	0.88 (2)
C10—C11	1.379 (4)	O2—H2	0.86 (2)
			
C6—C1—C2	119.6 (2)	C11—C12—C13	121.7 (3)
C6—C1—C7	120.4 (2)	C11—C12—H12	119.1
C2—C1—C7	120.0 (2)	C13—C12—H12	119.1
O1—C2—C1	122.8 (2)	C8—C13—C12	120.2 (3)
O1—C2—C3	118.3 (2)	C8—C13—H13	119.9
C1—C2—C3	118.9 (2)	C12—C13—H13	119.9
O2—C3—C4	119.8 (2)	C15—C14—C11	115.4 (3)
O2—C3—C2	120.2 (2)	C15—C14—H14A	108.4
C4—C3—C2	120.0 (2)	C11—C14—H14A	108.4
C3—C4—C5	121.1 (2)	C15—C14—H14B	108.4
C3—C4—H4	119.5	C11—C14—H14B	108.4
C5—C4—H4	119.5	H14A—C14—H14B	107.5
C6—C5—C4	120.0 (3)	C14—C15—C16	114.7 (3)
C6—C5—H5	120.0	C14—C15—H15A	108.6
C4—C5—H5	120.0	C16—C15—H15A	108.6
C5—C6—C1	120.4 (3)	C14—C15—H15B	108.6
C5—C6—H6	119.8	C16—C15—H15B	108.6
C1—C6—H6	119.8	H15A—C15—H15B	107.6
N1—C7—C1	121.3 (2)	C17—C16—C15	113.8 (3)
N1—C7—H7	119.3	C17—C16—H16A	108.8
C1—C7—H7	119.3	C15—C16—H16A	108.8
C13—C8—C9	118.5 (2)	C17—C16—H16B	108.8
C13—C8—N1	116.7 (2)	C15—C16—H16B	108.8
C9—C8—N1	124.8 (2)	H16A—C16—H16B	107.7
C10—C9—C8	120.2 (3)	C16—C17—H17A	109.5
C10—C9—H9	119.9	C16—C17—H17B	109.5
C8—C9—H9	119.9	H17A—C17—H17B	109.5
C9—C10—C11	121.9 (3)	C16—C17—H17C	109.5
C9—C10—H10	119.0	H17A—C17—H17C	109.5
C11—C10—H10	119.0	H17B—C17—H17C	109.5
C12—C11—C10	117.4 (2)	C7—N1—C8	124.0 (2)
C12—C11—C14	121.7 (3)	C2—O1—H1	104 (2)
C10—C11—C14	120.8 (3)	C3—O2—H2	105 (2)
			
C6—C1—C2—O1	−178.6 (2)	N1—C8—C9—C10	178.8 (3)
C7—C1—C2—O1	2.6 (4)	C8—C9—C10—C11	0.8 (5)
C6—C1—C2—C3	3.5 (4)	C9—C10—C11—C12	−0.5 (5)
C7—C1—C2—C3	−175.4 (2)	C9—C10—C11—C14	−177.4 (3)
O1—C2—C3—O2	−2.0 (4)	C10—C11—C12—C13	0.1 (5)
C1—C2—C3—O2	176.0 (2)	C14—C11—C12—C13	177.0 (3)
O1—C2—C3—C4	177.9 (2)	C9—C8—C13—C12	0.3 (4)
C1—C2—C3—C4	−4.1 (4)	N1—C8—C13—C12	−179.2 (2)
O2—C3—C4—C5	−177.8 (3)	C11—C12—C13—C8	0.0 (5)
C2—C3—C4—C5	2.4 (4)	C12—C11—C14—C15	128.0 (4)
C3—C4—C5—C6	0.0 (4)	C10—C11—C14—C15	−55.2 (4)
C4—C5—C6—C1	−0.6 (4)	C11—C14—C15—C16	−177.2 (3)
C2—C1—C6—C5	−1.2 (4)	C14—C15—C16—C17	179.6 (3)
C7—C1—C6—C5	177.6 (3)	C1—C7—N1—C8	176.5 (2)
C6—C1—C7—N1	178.9 (3)	C13—C8—N1—C7	−176.0 (3)
C2—C1—C7—N1	−2.2 (4)	C9—C8—N1—C7	4.6 (4)
C13—C8—C9—C10	−0.6 (4)		

### Hydrogen-bond geometry (Å, °)

**Table:**

*D*—H···*A*	*D*—H	H···*A*	*D*···*A*	*D*—H···*A*
O2—H2···O1	0.86 (2)	2.21 (3)	2.728 (2)	118 (3)
O2—H2···O1^i^	0.86 (2)	2.08 (3)	2.802 (3)	141 (3)
O1—H1···N1	0.88 (2)	1.74 (2)	2.555 (2)	155 (3)
C6—H6···Cg2^ii^	0.93	2.85	3.645 (3)	144
C10—H10···Cg1^ii^	0.93	2.80	3.491 (3)	132

Symmetry codes: (i) −*x*+1, −*y*+3, −*z*; (ii) −*x*+1, *y*−1/2, −*z*+1/2.

## References

[bb1] Bernstein, J., Davis, R. E., Shimoni, L. & Chang, N.-L. (1995). *Angew. Chem. Int. Ed. Engl.* **34**, 1555–1573.

[bb2] Calligaris, M., Nardin, G. & Randaccio, L. (1972). *Coord. Chem. Rev.* **7**, 385–403.

[bb3] Farrugia, L. J. (1997). *J. Appl. Cryst.* **30**, 565.

[bb4] Farrugia, L. J. (1999). *J. Appl. Cryst.* **32**, 837–838.

[bb5] Hadjoudis, E., Vitterakis, M. & Mavridis, I. M. (1987). *Tetrahedron*, **43**, 1345–1360.

[bb6] Koşar, B., Albayrak, C., Odabaşoğlu, M. & Büyükgüngör, O. (2005). *Acta Cryst.* E**61**, o2109–o2111.

[bb7] Lozier, R., Bogomolni, R. A. & Stoekenius, W. (1975). *Biophys. J.* **15**, 955–962. 10.1016/S0006-3495(75)85875-9 PMC1334761 1182271

[bb8] Maslen, H. S. & Waters, T. N. (1975). *Coord. Chem. Rev.* **17**, 137–176.

[bb9] Moustakali-Mavridis, I., Hadjoudis, B. & Mavridis, A. (1980). *Acta Cryst.* B**36**, 1126–1130.

[bb10] Sheldrick, G. M. (2008). *Acta Cryst.* A**64**, 112–122. 10.1107/S0108767307043930 18156677

[bb11] Stewart, J. M. & Lingafelter, E. C. (1959). *Acta Cryst.* **12**, 842–845.

[bb12] Stoe & Cie (2002). *X-AREA* and *X-RED32* Stoe & Cie, Darmstadt, Germany.

[bb13] Temel, E., Albayrak, Ç., Odabaşoğlu, M. & Büyükgüngör, O. (2007). *Acta Cryst.* E**63**, o2642. 10.1107/S0108270107003241 17339725

